# Blastoid Variant Mantle Cell Lymphoma with Complex Karyotype Including 11q Duplication

**DOI:** 10.4274/tjh.2012.0195

**Published:** 2014-09-05

**Authors:** Özge Özer, Selami K. Toprak, Enver Öte, Zerrin Yılmaz, Feride İffet Şahin

**Affiliations:** 1 Başkent University Faculty of Medicine, Department of Medical Genetics, Ankara, Turkey; 2 Başkent University Faculty of Medicine, Department of Hematology, Ankara, Turkey

**Keywords:** cytogenetics, non-Hodgkin’s lymphoma, Other lymphoproliferative diseases, Other leukemias

## Abstract

We describe a case of blastoid mantle cell lymphoma with a complex karyotype. The blastoid variant is a rare type of non-Hodgkin lymphoma exhibiting an aggressive clinical course. Mantle cell lymphoma is a distinct entity of mature B-cell neoplasms genetically characterized by the presence of t(11;14). In the present case, conventional analysis revealed structural abnormalities of chromosomes 2, 4, 6, 10, 13, and 19, along with 3 additional marker chromosomes. The derivative 1 chromosome determined in the case was a result of t(1p;11q). Our interesting finding was the presence of a different translocation between 11q and chromosome 1 in addition to t(11;14). Thus, the resulting 11q duplication was believed to additionally increase the enhanced expression of cyclin D1 gene, which is responsible in the pathogenesis of the disease. Fluorescence in situ hybridization method by the t(11;14) probe revealed clonal numerical abnormalities of chromosomes 11 and 14 in some cells. The detection of multiple abnormalities explains the bad prognosis in the present case. On the basis of our findings, we can easily conclude that results of cytogenetic analyses of similar mantle cell lymphoma patients would provide clues about new responsible gene regions and disease prognosis. In conclusion, it has been suggested that the presence of multiple chromosomal aberrations in addition to the specific t(11;14) may have a negative impact on clinical course and survival rate.

## OZET

Kompleks karyotipe sahip bir blastoid mantle hücreli lenfoma olgusunu sunuyoruz. Blastoid alt tip, saldırgan bir klinik seyre sahip olup, ender görülen bir Hodgkin dışı lenfoma alt tipidir. Mantle hücreli lenfoma ise, özellikle t(11;14) varlığı ile diğer B hücreli neoplazmlardan ayırt edilmektedir. Sunulan olguda geleneksel kromozom analizi sonrası 1, 2, 4, 6, 10, 13 ve 19. kromozomların yapısal anormalliklerine ek olarak 3 adet marker kromozom saptanmıştır. Hastamızda ayrıca, t(1p;11q) sonucunda oluşmuş türev 1. kromozom gözlenmiştir. Buradan hareketle, 11q bölgesinin, ilginç olarak t(11;14) haricinde 1. kromozom ile farklı bir translokasyona da katıldığını söyleyebiliriz. Bu nedenle ortaya çıkan 11q duplikasyonunun hastalığın oluşma sürecinden sorumlu tutulan siklin D1 geninin ifadelenmesini arttırdığı düşünülmektedir. Çalışma sırasında, floresan in situ melezleme yöntemi ile t(11;14) probu kullanılarak bazı hücrelerde klonal sayısal 11. ve 14. kromozom anormallikleri görüntülenmiştir. Çok sayıda kromozom bozukluğunun saptanmış olması olgumuzun kötü klinik gidişini açıklamaktadır. Hastamıza ilişkin bulgularımız ışığında, aynı tanıya sahip hasta grubunda yapılacak sitogenetik inceleme sonuçlarının yeni sorumlu gen bölgeleri hakkında ipuçları vereceği rahatlıkla söylenebilir. Sonuç olarak, hastamızda t(11;14) varlığına ek olarak çoklu kromozom bozukluklarının bulunmasının, klinik gidiş ve tedavi yanıtı üzerine olumsuz bir etki gösterdiği açıktır.

## INTRODUCTION

Mantle cell lymphoma (MCL) is a mature B-cell neoplasm defined as a distinct entity in 1992 constituting 4%-6% of all non-Hodgkin lymphomas [1]. The clinical course of MCL ranges from an indolent disease to a rapidly progressive malignancy, with poor prognosis and a median overall survival of about 3-5 years as reported in earlier studies [2,3]. The disease is typically advanced at diagnosis, with a leukemic component in 20%-30% of patients [1]. MCL is responsive to a great variety of initial therapies, but relatively short-term remissions are achieved with conventional strategies or with intensive treatment approaches [4]. The genetic hallmark of disease is the translocation t(11;14) (q13;q32) leading to aberrant expression of cyclin D1, which is not typically expressed in normal lymphocytes [1]. Many cases have additional cytogenetic abnormalities and often have a complex karyotype. Leukemic presentation of MCL can cause diagnostic confusion with other CD5+ B-cell leukemias and the use of cytogenetic techniques, together with morphology and immunophenotype, is required to establish the correct diagnosis [5]. Here, we report the clinical correlation and prognostic impact of a panel of cytogenetic abnormalities detected in a case of a blastoid variant of MCL with a rare association of a complex translocation by fluorescence in situ hybridization (FISH) and conventional cytogenetic analysis.

## CASE PRESENTATION

**Clinical Progression**

A 74-year-old female consulted with the Outpatient Clinic of the Gynecology and Obstetrics Department because of fatigue, abdominal pain, and B symptoms. Abdominal and thoracic computerized tomography revealed hepatosplenomegaly; paraaortic, mesenteric, and parailiac multiple enlarged lymph nodes; a mass lesion on the mid-line in accordance with conglomerate lymphoid tissue at the periumblical localization; and mediastinal, hilar, and bilateral axillary and supraclavicular widespread lymphadenopathies. Total excision biopsy was performed on the left supraclavicular lymph nodes and this biopsy material revealed diffuse infiltrations and patched nodular configurations, along with atypical irregularly shaped lymphoid cells that showed diffuse and strong immunohistochemical staining with cyclin D1 and CD20. The patient entered our close follow-up program with the diagnosis of MCL. Bone marrow biopsy material showed infiltration by flow cytometry and pathologic examination. The patient’s Mantle Cell Lymphoma International Prognostic Index score was 11 (high risk). During the course of diagnosis, the patient had febrile neutropenia and diffuse pleural effusion accompanying pneumonia. The patient was followed in the intensive care unit, with multiple antibiotic treatments againts pseudomonal, gram (+), fungal, and atypical agents in accordance with the febrile neutropenia protocol. At this time, an R-Hyper CVAD A-arm regimen containing cyclophosphamide, vincristine, doxorubicin, and dexamethasone alternating with a high-dose methotrexate and cytarabine + rituximab combination was started with modified doses. Active hematochezia started on the third day of chemotherapy, and in the following days, sepsis, disseminated intravascular coagulation, and multiple organ failure developed. On the sixth day of treatment, the patient died with the above-mentioned clinical picture despite all interventions.

**Genetic Analyses**

**Figure 1:**
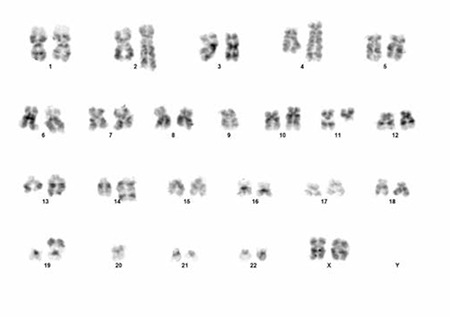
Karyotype of the patient showing t(11;14) and other chromosomal abnormalities 300x200 mm (96x96 DPI).

**Figure 2:**
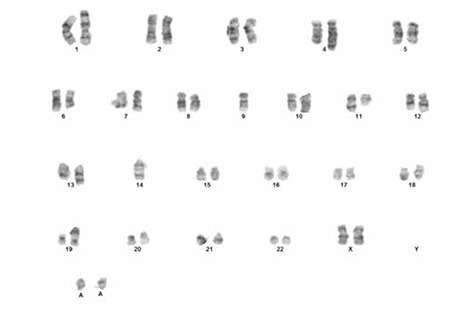
Karyotype including t(11;14) and other chromosomal abnormalities observed in the patient 374x275 mm (96x96 DPI).

Conventional cytogenetic analysis was performed on induced cultures set up from bone marrow aspiration samples. Chromosomes were harvested after 72 and 120 h of incubation following colcemid addition. Giemsa-trypsin (GTG) banded chromosomes were analyzed and the composite karyotype was reported according to the 2009 International System for Human Cytogenetic Nomenclature as 44x47, XX, der(1) t(1p;11q), der(2) t(2q;?) der(4) t(4q;11q), del(6q), der(10) t(10q;?), t(11;14) (q13;q32), der(13) t(13q;?), der(19) t(19p;?), +3mar[cp8]/46, XX[2] [6] (Figures 1 and 2).

For FISH, metaphase spreads were hybridized with the LSI IGH/CCND1-XT DF probe (Vysis, Abbott Park, IL, USA) in order to investigate MCL-specific t(11;14) translocation. Additionally, numerical abnormalities of chromosomes 11 and 14 were also observed.

**Figure 3:**
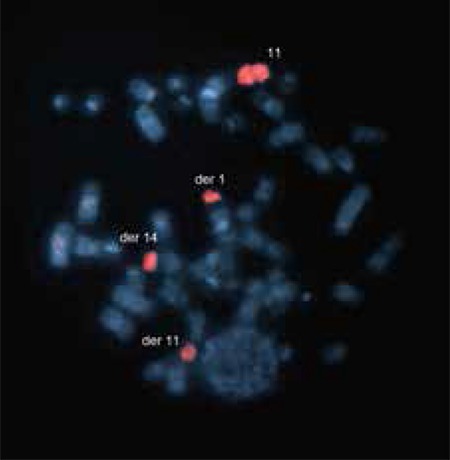
FISH result of the patient after hybridization with WCP 11 probe. Signals were observed on der(1) t(1p;11q), der(11) t(11;14), (der)14 t(11;14) and on normal chromosome 11 on a previously GTG banded metaphase 364x270 mm (96x96 DPI).

Hybridization with the whole chromosome 11 probe (WCP 11, ID Labs, London, ON, Canada) supported our finding in the conventional karyotype, as we observed the chromosome 11 signal on chromosome 1, indicating a translocation between chromosomes 1 and 11 (Figure 3).

## DISCUSSION AND REVIEW OF THE LITERATURE

Although MCL accounts for only 5% to 10% of all lymphoma diagnoses, it contributes disproportionately to lymphoma deaths because of its often aggressive clinical course and lack of definitive curative therapy. MCL is a malignancy with distinct molecular genetics and pathological features. Peripheral blood involvement has been reported with variable frequency, but information on the natural history of patients presenting with leukemia is lacking [7]. For the present case, diagnosis was based on morphology, immunophenotype, presence of t(11;14), histology, and cyclin D1 expression. MCL usually consists of small to medium-sized lymphoid cells with irregular nuclear contours, somewhat dispersed chromatin, inconspicuous nucleoli, and scant cytoplasm [8]. According to the World Health Organization lymphoma classification, 4 cytologic variants of MCL can be defined, including the small cell variant, the marginal zone-like variant, the blastoid variant, and the pleomorphic variant [9]. The blastoid (approximately 20% of all MCLs) and pleomorphic variants are considered to be associated with a poorer prognosis.

The patient presented with splenomegaly, abdominal and mediastinal lymphadenopathy, pleural effusion, and peripheral blood lymphoma cells. A bone marrow (BM) smear showed an increase in large, abnormal lymphoid cells with oval or round nuclei, distinct nucleoli, and basophilic cytoplasm with vacuolization. Flow cytometry analysis from peripheral blood and BM showed these cells to be positive for CD5, CD19, IgM/D, and CD20 and negative for CD10 and CD23. Additionally, an immunohistochemical pattern positive for CD5, CD19, bcl-2, CD20, CD79a, and cyclin D1 and negative for CD23 and CD30 was found in both lymph node and BM specimens. A blastoid variant of MCL was diagnosed from these results. According to the Ann Arbor staging system, the patient was accepted as stage IV and the performance status was grade 3-4 according to the Eastern Cooperative Oncology Group scale.

The CCND1/IGH gene rearrangement is the most important factor in the diagnosis of MCL. The t(11;14) determines the ectopic and deregulated expression of cyclin D1, which is not typically expressed in normal lymphoid cells. A disease’s aggressive clinical behavior is related to its genetic and molecular pathogenesis, which integrates alterations in cell cycle regulation, DNA damage response mechanisms, and activation of cell survival pathways [10].

The patient died during first-line therapy with a rapid and bad course, in accordance with the aggressive properties of primary disease with a complex karyotype, along with the contribution of her advanced age and chemotherapeutic side effects. The presence of multiple chromosome abnormalities in addition to t(11;14) is well known to be associated with blastoid variants and poor prognoses in MCL [11]. The additional chromosomal abnormality determined in the present case as a result of conventional cytogenetic analysis was reported in previous studies in different cases [12,13]. Espinet et al., in their study including 145 MCL cases, reported a total of 550 chromosomal changes (14 numerical and 403 structural). Among these, the most common secondary abnormalities were loss of 1p, 13q, and 17p; changes in 10p; and gains in 3q [13]. Numerical abnormalities include trisomy 3, trisomy 12, monosomy 8, monosomy 9, and loss of sex chromosomes (X Y). The most common secondary abnormality was 13q deletion in the classic variant of MCL. In the present case, conventional analysis revealed structural abnormalities of chromosomes 2, 4, 6, 10, 13, and 19, along with additional 3 marker chromosomes. The derivative 1 chromosome determined in the case was as a result of t(1p;11q). Our interesting finding was the presence of a different translocation between 11q and chromosome 1 in addition to t(11;14). Thus, the resulting 11q duplication was believed to additionally increase the enhanced expression of the cyclin D1 (CCND1) gene, which is responsible in the pathogenesis of the disease. We think that this finding increases the importance of the current case. Moreover, the interest of this case lies in the presence of these aberrant clones that are not very widely known or studied although their presence was pointed out many years ago. Tetraploid chromosome clones in blastoid MCL variants with chromosome 1 involvement were first reported by Ott et al., while recurrent losses at 1p (55%), 8p (29%), 9q (29%), 11q (55%), 13q (42%), and 17p (32%) and gains at 3q (39%), 8q (26%), 15q (23%), and 18q (23%) were recently reported in aggressive MCL presenting genomic complexity and high proliferation index [14,15].

Previous studies have reported that, in similar situations, additional findings can be determined by the FISH method in addition to conventional cytogenetics and that t(11;14) alone is not sufficient for development of a malignant phenotype [12]. Thus, the use of the FISH method in addition to conventional cytogenetic analysis for diagnosis would be beneficial for the patient in terms of clinical follow-up and treatment. In the present case, the FISH method by the t(11;14) probe revealed clonal numerical abnormalities of chromosomes 11 and 14 in some cells. Although the FISH method does not require metaphase spreads, determining signals on the same metaphases, especially following GTG banding, would contribute to the clarification of the disease pathogenesis. However, the success of hybridization in the FISH method performed following GTG banding is low. In the present case, we were not able to evaluate t(4q;11q) due to hybridization failure after GTG banding.

In cases with more than 2 structural abnormalities, the clinical prognosis has been reported to be unfavorable. In the present case, multiple structural abnormalities were detected concurrently. Following the analysis of the 10 metaphases examined, a complex karyotype showing clonal abnormalities was reported. The detection of multiple abnormalities explains the bad prognosis in the present case. Although the enhanced expression of the CCND1 gene alone is held responsible for the development of lymphoma, the additional influence of tumor suppressors like TP53 and RB1 and oncogenes like BCL2, CD4, and MYC play a role in the development of MCL [12]. On the basis of our findings in the current case, we can easily conclude that results of cytogenetic analyses of similar MCL patients would provide clues about new responsible gene regions and disease prognosis.

We did not have the opportunity to investigate the present case at the molecular level. However, we think that in cases representing a complex karyotype, imaging with contemporary molecular methods that enable detailed investigation of all chromosomes and related gene areas with a single hybridization of patient DNA, such as array CGH, would be advantageous in clarifying the mechanisms underlying disease pathogenesis.

In conclusion, it has been suggested that the presence of multiple chromosomal aberrations in addition to the specific t(11;14) may have a negative impact on clinical course and survival rate. Written informed consent was obtained from the patient’s family.

**Conflict of interest statement**

None of the authors of this paper has a conflict of interest, including specific financial interests, relationships, and/or affiliations relevant to the subject matter or materials included.
